# Occupational Reproductive Health Risks Among Women Healthcare Workers: A Narrative Review for Clinical Surveillance, Preconception Counseling, and Prevention

**DOI:** 10.3390/jcm15124651

**Published:** 2026-06-15

**Authors:** Oh-Hyun Kwon, Gyu-Jin Sim, Sun-Haeng Choi

**Affiliations:** 1 Graduate School of Safety Engineering, Seoul National University of Science and Technology, Seoul 01811, Republic of Korea; ohyun1017@kfsi.or.kr (O.-H.K.); ssim0111@gmail.com (G.-J.S.); 2 Department of Occupational and Environmental Medicine, College of Medicine, Chungbuk National University, Cheongju 28644, Republic of Korea; 3 Department of Occupational and Environmental Medicine, Chungbuk National University Hospital, Cheongju 28644, Republic of Korea

**Keywords:** women healthcare workers, occupational reproductive hazards, antineoplastic agents, high-level disinfectants, clinical surveillance, reproductive medicine, occupational medicine, miscarriage, preconception counseling, hazardous drugs

## Abstract

**Background/Objectives**: Despite well-documented chemical and physical hazards in healthcare settings, existing reviews of occupational reproductive risks have largely focused on single-agent risk estimation and have rarely translated occupational hygiene evidence into clinical decision-making frameworks for reproductive counseling and surveillance. This narrative review synthesizes evidence across multiple occupational exposure categories—antineoplastic agents, high-level disinfectants (HLDs), sterilants, and work-organization factors—and proposes an integrated, clinically operational framework for preconception counseling, pregnancy-sensitive risk stratification, exposure-control verification, and reproductive health surveillance among women healthcare workers. **Methods**: A structured narrative literature search was conducted across PubMed/MEDLINE, Scopus, Web of Science, and Embase from database inception through January 2025 and updated in March 2026. The review was guided by a Population–Exposure–Comparison–Outcome (PECO) framework and structured using Search–Appraisal–Synthesis–Analysis (SALSA) principles and the Scale for the Assessment of Narrative Review Articles (SANRA). Evidence quality was summarized using a modified hierarchy-of-evidence classification provided as a reader aid. This narrative review employed structured transparency tools but does not claim the methodological status of a systematic review. Quantitative meta-analytic pooling was not performed owing to substantial heterogeneity across study designs, exposure assessment methods, and outcome definitions; findings were synthesized narratively by exposure category. **Results**: The strongest and most consistent evidence was identified for occupational exposure to antineoplastic agents, which has been associated with spontaneous abortion, stillbirth, congenital abnormalities, impaired fecundability, and selected cancer-related concerns. HLDs and sterilants represent exposure categories warranting precautionary attention, with some evidence suggesting possible adverse effects on fecundability and early pregnancy maintenance; however, findings are considerably more heterogeneous, context-dependent, and reliant on self-reported exposure assessment than those for antineoplastic agents. Broader workplace factors, including shift work, prolonged working hours, physical workload, and mixed exposures, may further contribute to reproductive risk. The synthesis supports task-specific occupational history taking, exposure-control verification, and pregnancy-sensitive risk stratification. **Conclusions**: This review provides a multi-exposure, clinically operational framework that bridges occupational hygiene evidence with reproductive healthcare delivery, offering practical decision-support tools for clinicians managing women healthcare workers during preconception, pregnancy, and lactation. The framework includes structured occupational history-taking questions, a clinical decision pathway with evidence-tier classification, and a prevention matrix linking exposure sources to workplace controls and clinical actions. Integrating task-specific occupational history taking into routine reproductive care may improve detection of preventable workplace risks and support timely accommodation, while clinicians should calibrate recommendation strength to the underlying evidence quality for each exposure category.

## 1. Introduction

Healthcare settings are commonly perceived as environments for healing and diagnosis; however, they are also highly complex occupational environments where workers encounter physical, ergonomic, psychosocial, biological, and chemical hazards. This issue holds particular clinical significance for women healthcare workers, as nursing, pharmacy, medical, technical, and laboratory-related occupations include large numbers of women and often overlap with reproductive-age employment [1,2,3,4]. In hospitals, clinics, oncology units, endoscopy suites, central sterile processing departments, and pathology laboratories, women may be exposed to hazardous chemicals, including antineoplastic agents, high-level disinfectants, sterilants, formaldehyde, ethylene oxide, solvents, anesthetic gases, and other agents of reproductive or carcinogenic concern [5,6,7,8,9,10,11,12,13].

These exposures are clinically relevant because adverse reproductive outcomes associated with occupational hazards may include delayed time to pregnancy, spontaneous abortion, stillbirth, preterm birth, low birth weight, intrauterine growth restriction, congenital abnormalities, and shortened breastfeeding duration [1,2,4,5,7,14,15,16,17,18,19,20,21,22]. Recent evidence suggests that these reproductive and pregnancy outcomes are linked not only to direct chemical exposure but also to broader work-organization factors, including prolonged working hours, shift work, night work, physical workload, and psychosocial stress [1,2,4,19,20,21,22]. Accordingly, reproductive health in healthcare workers should be treated as an integrated occupational and clinical health concern rather than solely an individual obstetric issue.

Despite these concerns, comprehensive occupational history taking is frequently overlooked in routine gynecological, preconception, and prenatal care. Antineoplastic agents have received the greatest attention because many are hazardous drugs with genotoxic, mutagenic, carcinogenic, teratogenic, or reproductive-toxic properties [9,10,11,14,17,23,24]. The current NIOSH hazardous-drug framework and USP General Chapter <800> provide practical approaches for hazard identification and safe handling in healthcare settings [3,10,24,25]. At the same time, high-level disinfectants and sterilants, including glutaraldehyde, ortho-phthalaldehyde (OPA), peracetic acid, ethylene oxide, and formaldehyde, represent less visible but clinically relevant exposures in device reprocessing, sterilization, endoscopy, and pathology settings [5,6,7,8,26,27,28,29].

Several systematic reviews and meta-analyses have examined the reproductive effects of antineoplastic agents or specific chemical exposures in isolation [14,17,18,27]. However, no review to date has simultaneously addressed multiple occupational exposure categories—including antineoplastic agents, HLDs, sterilants, and work-organization factors—within an integrated clinical decision-making framework designed for reproductive health surveillance. Existing reviews have primarily focused on risk estimation (e.g., pooled odds ratios for spontaneous abortion), whereas the practical clinical challenge lies in translating heterogeneous epidemiological evidence into actionable guidance for occupational history taking, risk stratification, and workplace accommodation during preconception, pregnancy, and lactation. Moreover, the substantial heterogeneity across the available evidence base—arising from differences in exposure assessment methods (e.g., job title versus biomonitoring versus task-based estimation), outcome definitions (e.g., clinically confirmed versus self-reported miscarriage), study populations (e.g., oncology nurses versus general hospital staff), and study designs (e.g., prospective cohort versus retrospective survey)—renders quantitative meta-analytic pooling across all exposure categories methodologically inappropriate and clinically misleading [2,14,17,18,30]. A narrative synthesis that transparently maps evidence patterns by exposure category and clinical context is therefore better suited to inform clinical practice.

Therefore, this narrative review aims to synthesize current evidence on occupational reproductive hazards among women healthcare workers and to translate this evidence into a clinically applicable framework for occupational history taking, preconception counseling, pregnancy-sensitive risk stratification, exposure-control verification, and reproductive health surveillance (Box 1).

Box 1 **What This Review Adds.**

1. This review integrates evidence on antineoplastic agents, high-level disinfectants, sterilants, and work-organization factors rather than focusing on a single exposure category.
2. It translates heterogeneous occupational and reproductive health evidence into a clinically usable framework for preconception counseling, pregnancy-sensitive risk stratification, and workplace accommodation.
3. It provides practical clinical questions for occupational history taking and exposure-control verification among women healthcare workers.
4. It highlights evidence gaps for non-nursing healthcare workers, including sterilization technicians, pathology staff, laboratory personnel, and environmental services workers.

## 2. Narrative Review Methodology

### 2.1. Search Strategy

As a narrative review, this study does not claim the methodological rigor of a systematic review or meta-analysis. The PECO framework, adapted PRISMA-style flow diagram, and SANRA self-assessment were adopted as transparency-enhancing tools to improve reproducibility and reader confidence, not as indicators of formal systematic appraisal. Readers should interpret all findings within the inherent limitations of narrative synthesis, including non-exhaustive literature coverage, absence of formal risk-of-bias scoring, and the predominantly observational, retrospective, and self-report-dependent nature of the underlying evidence base.

A structured narrative literature search was conducted across PubMed/MEDLINE, Scopus, Web of Science, and Embase from database inception through January 2025 and updated in March 2026 to identify newly published studies and guideline documents. Searches emphasized peer-reviewed literature on women healthcare workers, nurses, pharmacists, laboratory personnel, and hospital staff exposed to antineoplastic agents, hazardous drugs, HLDs, sterilants, formaldehyde, ethylene oxide, and related reproductive or cancer-related outcomes. Guideline and policy documents from NIOSH, USP, CDC/NIOSH, ASHP, and the Cochrane Collaboration were also reviewed to support clinical and occupational prevention recommendations [3,10,24,25,31]. The database-specific search strategies, including search strings and filters, are provided in Supplementary Table S2.

### 2.2. Search Terms

The search strategy used Boolean combinations of population terms (“healthcare workers”, “nurses”, “pharmacists”, “laboratory workers”, “hospital staff”), exposure terms (“antineoplastic agents”, “hazardous drugs”, “high-level disinfectants”, “sterilants”, “formaldehyde”, “ethylene oxide”, “closed-system transfer devices”), and outcome terms (“reproductive outcomes”, “miscarriage”, “spontaneous abortion”, “stillbirth”, “fecundity”, “infertility”, “congenital abnormalities”, “pregnancy outcomes”, “occupational cancer”).

### 2.3. PECO Framework

To ensure systematic eligibility assessment, literature selection was guided by a Population–Exposure–Comparison–Outcome (PECO) framework [30,32]. Although PECO frameworks are most commonly associated with systematic reviews, their explicit use in narrative reviews has been recommended to enhance transparency and reproducibility of the evidence identification process. Table 1 summarizes the PECO criteria applied in this review.

### 2.4. Eligibility Criteria and Literature Selection

Recent articles published from 2018 onward were prioritized to reflect current workplace practices, but older landmark studies were included when they provided foundational epidemiological evidence or widely cited guidance directly relevant to the review question [4,12,16,17,18,19,20,23,33,34,35,36,37]. Included evidence comprised prospective and retrospective cohort studies, cross-sectional studies, case–control studies, systematic reviews, meta-analyses, scoping reviews, narrative reviews, biological monitoring studies, and major occupational safety guidelines. Articles were excluded when they did not meet the PECO criteria (Table 1), were not peer-reviewed, lacked transparent methodological reporting, or addressed only general environmental exposure without occupational relevance.

In total, 320 records were identified through database searching and 45 additional records were identified through guideline repositories and reference list scanning. After removal of 80 duplicates, 285 records were screened by title and abstract, of which 200 were excluded as irrelevant. A total of 85 full-text articles were assessed for eligibility against the PECO criteria; 36 were excluded (12 lacked an occupational focus, 8 were non-peer-reviewed, 9 reported no reproductive outcome, and 7 had insufficient methodological reporting). A total of 49 sources were included in the final narrative synthesis.

**Figure 1:**
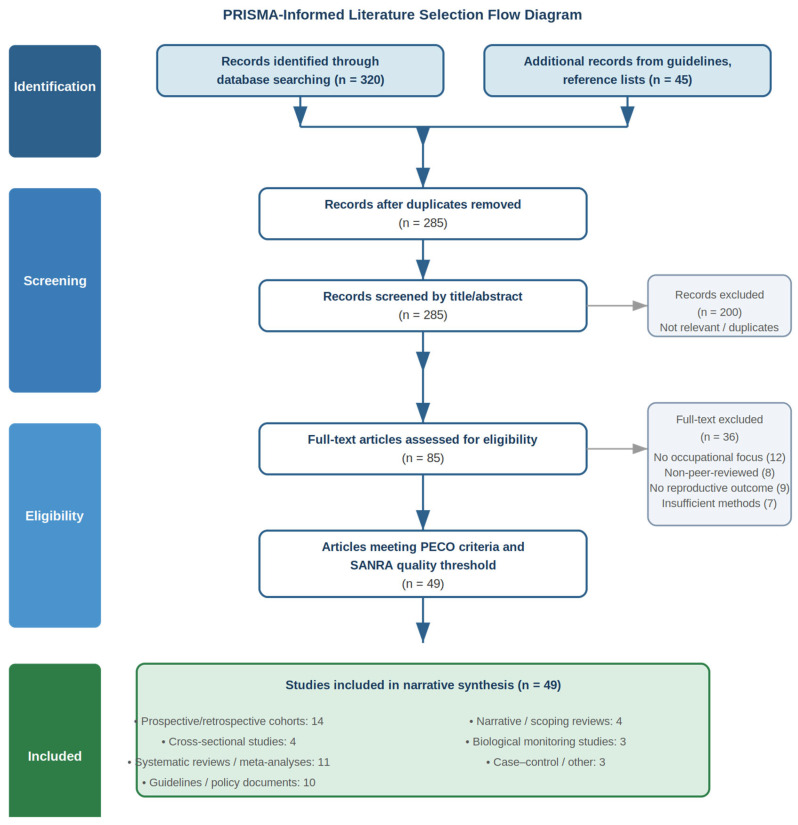
Adapted PRISMA-style flow diagram illustrating the literature selection process. This diagram is provided as a transparency aid for this narrative review and does not imply systematic review methodology. Adapted from the PRISMA 2020 flow diagram (Page et al., 2021) [38]. PECO, Population–Exposure–Comparison–Outcome; SANRA, Scale for the Assessment of Narrative Review Articles.

An adapted PRISMA-style flow diagram, used here as a transparency aid rather than as a claim of systematic methodology, is presented in Figure 1.

### 2.5. Evidence Synthesis Framework

The synthesis was organized according to exposure category and clinical relevance rather than by quantitative pooling. To improve transparency and methodological rigor, the review was structured using SANRA principles and a SALSA-informed approach [30,32,38,39]. A self-assessment against the six SANRA items is provided in Supplementary Table S1.

This narrative review was not registered in PROSPERO (International Prospective Register of Systematic Reviews), as PROSPERO accepts registrations for systematic reviews and does not register narrative reviews (Centre for Reviews and Dissemination, University of York). The decision to conduct a narrative rather than systematic review was methodologically grounded in the substantial heterogeneity across the included evidence base, as discussed below.

A formal quantitative meta-analysis was not performed for three principal reasons. First, the included primary studies employed heterogeneous exposure assessment methods, ranging from job-title classification and self-reported task inventories to dermal wipe sampling and urinary biomonitoring, making it methodologically inappropriate to combine effect estimates across studies without introducing substantial clinical and statistical bias. Second, outcome definitions varied considerably: some studies used clinically confirmed pregnancy loss, whereas others relied on self-reported miscarriage or registry-based coding, each with different sensitivity and specificity [2,7,14,17]. Third, the review scope intentionally spans multiple exposure categories (antineoplastic agents, HLDs, sterilants, work-organization factors) and multiple outcome domains (fecundability, miscarriage, congenital abnormalities, preterm birth, cancer risk), which represent distinct biological pathways and risk profiles; pooling across these categories would obscure clinically meaningful differences. Where meta-analytic evidence already exists for specific agent–outcome pairs (e.g., antineoplastic agents and spontaneous abortion [14,18]), we cite and discuss those pooled estimates rather than duplicating quantitative synthesis.

### 2.6. Evidence Quality Assessment

Because the purpose of this review was not to generate pooled causal estimates but to translate heterogeneous epidemiological, toxicological, and guideline-based evidence into clinically usable decision support, we used structured evidence mapping rather than formal risk-of-bias scoring. To improve transparency, each key study was summarized according to study design, exposure assessment method, outcome definition, effect estimate, evidence level, and principal limitations (Table 2). To aid reader interpretation, evidence levels were classified using a modified hierarchy loosely adapted from the Oxford Centre for Evidence-Based Medicine (OCEBM) criteria [40]. These classifications are provided as a descriptive reader aid and do not constitute formal quality appraisal:

Level I denotes systematic reviews or meta-analyses of well-designed studies; Level II denotes individual prospective cohort studies; Level III denotes retrospective cohort or case–control studies; Level IV denotes cross-sectional studies or case series; and Level V denotes narrative reviews, expert opinion, or guideline documents without original data.

This approach enables readers to distinguish between findings derived from prospective cohort evidence (e.g., Nurses’ Health Study 3 analyses [5,7,41,42]) and those based on cross-sectional surveys or expert consensus, without implying that a formal systematic quality appraisal was performed. The evidence-level assignments are presented transparently as a reader aid rather than as a substitute for formal risk-of-bias assessment.

## 3. Review of Occupational Exposures and Clinical Outcomes

### 3.1. The Clinical Relevance of Reproductive Risk in Healthcare Settings

The overlap between reproductive-age employment and healthcare work creates an important clinical and occupational medicine issue. Women healthcare workers may perform tasks involving hazardous drug preparation or administration, device reprocessing, sterilization, pathology procedures, laboratory handling, shift work, prolonged standing, heavy lifting, and demanding patient-care activities [1,2,4,5,7,19,20,21,22]. These exposures may be particularly relevant during preconception, implantation, early pregnancy, organogenesis, and lactation. However, the extent to which low-level occupational exposures translate into clinically measurable reproductive outcomes depends on exposure intensity, frequency, timing, route, cumulative duration, and the effectiveness of control measures [7,12,14,16,17,18,31,41,42].

Recent studies indicate that reproductive outcomes among healthcare workers are influenced not only by single-agent chemical exposure but also by broader work-organization factors. Izadi et al. reported that chemical exposures, prolonged working hours, shift work, and psychiatric factors were associated with selected reproductive and pregnancy-related outcomes among women healthcare workers [1]. Marsters et al. also found that evidence on physician-related and healthcare-related occupational hazards is extensive but heterogeneous, with some data suggesting elevated miscarriage risk among healthcare workers and possible associations between long work hours and miscarriage or preterm birth [2]. These findings support task-level occupational history taking (see Section 4.2).

The following sections are organized in order of decreasing evidence strength. Antineoplastic agents (Section 3.2) represent the strongest and most consistent evidence domain, supported by prospective cohorts, meta-analyses, and established biological plausibility. HLDs and sterilants (Section 3.3) occupy an intermediate position with heterogeneous but clinically relevant signals. Work-organization factors (Section 3.4) are discussed more concisely as contributing risk modifiers supported primarily by cross-sectional and observational evidence.

### 3.2. Antineoplastic Agents: Reproductive and Cancer-Related Clinical Concerns

#### 3.2.1. Mechanisms and Biological Plausibility

Antineoplastic agents represent the most extensively studied occupational reproductive hazard in healthcare settings. Many agents interfere with DNA synthesis, chromosomal integrity, or cell division, which provides biological plausibility for reproductive and developmental concerns when occupational exposure occurs during sensitive reproductive periods [9,17,23,37,43,44]. Cytogenetic evidence, including chromosomal aberrations and sister chromatid exchange, supports the possibility that workplace exposure can produce measurable biological effects in exposed healthcare workers, although biomarker findings do not by themselves establish clinical reproductive causality [43,44].

Healthcare workers may be exposed during preparation, administration, disposal, spill management, handling of contaminated intravenous tubing, surface contact, and contact with patient excreta [9,10,11,17,23,33,36]. Dermal exposure appears particularly important because surface contamination can persist even where formal compounding controls are present [11,18,35,36,45,46]. Biological monitoring studies also show that urine-based assessments can detect occupational contamination in some settings, supporting the value of environmental and biological surveillance when feasible [11,47,48].

#### 3.2.2. Epidemiological Evidence

The earliest evidence suggested a reproductive risk signal among nurses who prepared or handled cytostatic or antineoplastic drugs. Stucker et al. observed a higher frequency of spontaneous abortion among exposed nurses than among unexposed nurses [34], and Valanis et al. reported an increased risk of spontaneous abortion among nurses and pharmacists exposed to antineoplastic agents during pregnancy [23]. Fransman et al. later used task-based dermal exposure estimates and reported that higher exposure was associated with longer time to pregnancy and increased risks of premature delivery and low birth weight [35].

More recent evidence is nuanced but clinically important. Lawson et al. reported an association between antineoplastic exposure and spontaneous abortion in the Nurses’ Health Study II [16]. Nassan et al. found that prepregnancy handling of antineoplastic drugs showed miscarriage risk signals, particularly relevant to the preconception window [42]. In contrast, Nassan et al. also reported that antineoplastic drug administration was not strongly associated with reduced fecundity in a setting where exposure controls were widely used [41]. This apparent inconsistency is clinically informative: it suggests that exposure intensity, route, timing, and control effectiveness may modify risk rather than exposure presence alone [24,31,41,42].

Meta-analytic evidence supports the clinical relevance of this concern. Liu et al. concluded that occupational exposure to antineoplastic agents among nurses was associated with spontaneous abortion, stillbirth, and congenital abnormalities [14]. Connor et al. similarly reviewed reproductive risks among workers handling antineoplastic drugs and concluded that chronic low-level exposure appears to be associated with increased adverse reproductive outcomes, although study limitations and heterogeneity remain important [17]. These findings support a precautionary clinical approach, particularly for workers who are trying to conceive, pregnant, undergoing fertility treatment, or breastfeeding.

It should be noted, however, that the majority of these studies relied on self-reported exposure assessment, retrospective pregnancy recall, and job-title-based or department-level exposure classification, which may introduce non-differential misclassification and either attenuate or inflate risk estimates. Only Fransman et al. (2007) [35] employed task-based dermal exposure modeling, while most other studies—including the Nurses’ Health Study II and III analyses—relied on self-reported occupational exposure. Publication bias may also overrepresent positive findings, as studies reporting null associations may be less likely to be published.

### 3.3. High-Level Disinfectants and Sterilants

#### 3.3.1. Related but Distinct Exposure Categories

High-level disinfectants and sterilants are related but distinct exposure categories. HLDs are commonly used for reprocessing semi-critical medical devices, whereas sterilants such as ethylene oxide or formaldehyde may be used in sterilization, pathology, anatomy, or laboratory-related settings [5,6,7,8,26,27,28,29]. Because these agents differ in chemistry, volatility, reactivity, and toxicological profile, they should not be treated as a single uniform exposure. Instead, they should be considered together as healthcare-related disinfection and sterilization exposures that require task-level assessment.

Gaskins et al. showed that occupational use of HLDs was associated with longer time to pregnancy among nurses attempting conception, and that protective equipment appeared to attenuate this association [5]. Ding et al. found no overall association between HLD use and miscarriage but observed an increased risk signal when analyses were restricted to pregnancies occurring within 12 months of baseline exposure [7]. Jiang et al. reported associations between maternal occupational exposure and adverse pregnancy outcomes among Chinese nurses, including signals involving disinfectants and anticancer drugs [15]. Together, these findings suggest that exposure timing, outcome window, and measurement approach are central to interpretation.

Compared with antineoplastic agents, evidence for HLDs and sterilants remains substantially less consistent, more dependent on exposure timing, agent type, task characteristics, and control measures, and predominantly derived from self-reported exposure assessment without quantitative dose characterization. The absence of quantitative exposure data (e.g., air monitoring or biological monitoring) in most HLD studies limits the precision of risk estimation and precludes dose–response analysis. The healthy worker effect may further attenuate observed associations, as workers experiencing adverse reproductive outcomes may selectively leave exposed positions. Therefore, clinical recommendations for HLD-exposed workers should be classified as Tier 2 (precautionary) and should emphasize exposure-control verification and individualized counseling rather than generalized work restriction.

#### 3.3.2. Formaldehyde and Ethylene Oxide

Formaldehyde and ethylene oxide require particular attention in pathology, anatomy, sterilization, and device-reprocessing environments. Foroughi et al. reported formaldehyde exposure and carcinogenic/non-carcinogenic risk concerns in hospital pathology laboratories [6]. Protano et al. summarized carcinogenic effects of occupational formaldehyde exposure [8], while Duong et al. reviewed reproductive and developmental toxicity of formaldehyde and reported increased risks in meta-analysis, while emphasizing potential bias and the need for quantitative exposure assessment [27]. Xu et al. found higher plasma formaldehyde concentrations among women with miscarriage in a Chinese case–control study, although this was not limited to healthcare workers [26].

Ethylene oxide is used in some sterilization contexts and is a recognized carcinogenic hazard. Gresie-Brusin et al. reported increased risks of spontaneous abortion and pregnancy loss among hospital sterilizing-unit workers highly exposed to ethylene oxide [28]. NIOSH reproductive-health guidance also identifies formaldehyde as a workplace exposure that may increase the risk of fertility problems or miscarriage and highlights pathology and anatomy laboratory workers as potentially exposed groups [29]. These sources support careful exposure-control verification rather than broad generalization across all HLDs or sterilants.

### 3.4. Mixed and Work-Organization-Related Reproductive Risks

Reproductive risk among women healthcare workers should not be interpreted solely through single-agent chemical exposure. Healthcare work often involves shift work, night work, prolonged standing, patient handling, lifting, sleep disruption, workplace stress, and chemical mixtures. Systematic reviews and meta-analyses suggest that associations between shift work, long working hours, physical workload, and adverse pregnancy outcomes are generally modest and heterogeneous, but they remain clinically relevant when combined with chemical exposure or previous reproductive risk factors [2,4,19,20,21,22].

It should be noted that nearly all evidence in this domain derives from cross-sectional or retrospective designs with self-reported exposure and outcome assessment, creating substantial vulnerability to recall bias, temporal ambiguity, and residual confounding by unmeasured lifestyle, socioeconomic, and health behavior factors. These associations, while biologically plausible, should be interpreted as hypothesis-generating rather than as a basis for definitive clinical recommendations and are classified as Tier 3 (expert opinion) in our evidence framework.

Meta-analytic evidence suggests that associations between shift work, prolonged working hours, physical workload, and adverse pregnancy outcomes are generally modest and heterogeneous [19,20,21,22]. Risks from shift work alone appear small but not absent, while occupational physical demands require task-specific rather than job-title-based evaluation [19,20,21]. Results remain inconsistent across studies, and most evidence is derived from observational designs with self-reported exposures (Tier 3).

Clinically, this supports whole-work assessment: a healthcare worker’s reproductive risk profile should integrate chemical tasks, work schedule, physical demands, and psychological stress.

## 4. Clinical Implications for Occupational and Reproductive Medicine

### 4.1. International Guidelines as a Clinical Tool

Clinicians can use established occupational safety frameworks to support exposure-control verification and workplace accommodation discussions. The NIOSH List of Hazardous Drugs in Healthcare Settings, 2024, is designed to help healthcare employers identify hazardous drugs and supersedes the 2016 NIOSH list [10]. USP General Chapter <800> provides standards for safe handling of hazardous drugs to minimize exposure among healthcare personnel, patients, and the environment [3]. ASHP guidelines provide additional practice-oriented recommendations for receiving, compounding, administering, transporting, and disposing of hazardous drugs [24].

In settings where USP <800>, NIOSH, ASHP, or comparable standards are applied, clinicians should use these frameworks to support risk communication rather than making generalized work prohibitions. Cochrane evidence on closed-system drug-transfer devices (CSTDs) indicates that certainty regarding exposure reduction remains low, but CSTDs remain important as part of a hierarchy of controls and broader safe-handling program [31]. Therefore, clinical recommendations should be tied to actual tasks, exposure controls, and reproductive timing.

To assist clinicians in calibrating the strength of their recommendations to the strength of the underlying evidence, we propose a three-tier classification: Tier 1 (Evidence-supported): recommendations backed by converging prospective cohorts, meta-analyses, and established biological plausibility, principally applicable to antineoplastic agents; Tier 2 (Precautionary): recommendations for exposures with biological plausibility but heterogeneous or context-dependent epidemiological evidence, applicable to HLDs and specific sterilants; Tier 3 (Expert opinion): recommendations based primarily on cross-sectional, survey-based, or limited observational evidence, applicable to mixed exposures and work-organization factors. This classification is applied throughout the remaining clinical sections, figures, and tables.

### 4.2. Occupational History Taking and Clinical Risk Stratification

As outlined in the Introduction, integrating occupational assessment into reproductive care is essential. The cornerstone of this integration is structured occupational history taking, as standard intake forms often capture only job title. Clinicians should ask about drug preparation, administration, disposal, spill cleanup, surface contamination, HLD and sterilant use, laboratory/pathology tasks, exposure controls, work schedule, pregnancy status, fertility treatment, prior miscarriage, and lactation.

#### Exposure Assessment Considerations for Clinical Practice

The heterogeneity in exposure assessment methods across the underlying evidence base has direct implications for clinical practice. For clinicians conducting occupational history taking, the following hierarchy of exposure characterization approaches may be considered in order of decreasing clinical informativeness:

First, task-based assessment represents the most informative and clinically feasible approach: asking specifically about drug preparation, administration, spill cleanup, device reprocessing, sterilization tasks, and pathology procedures, rather than relying on job title alone. Job title (e.g., ‘nurse’ or ‘pharmacist’) is insufficient because exposure intensity varies substantially by specific tasks performed, workplace control measures in place, and institutional safety culture.

Second, exposure-control verification—whether CSTDs, biological safety cabinets, local exhaust ventilation, appropriate gloves, and spill protocols are used consistently and correctly—provides critical context for risk stratification, as several studies have demonstrated that consistent use of exposure controls attenuates reproductive risk signals [5,31,41].

Third, temporal characterization—frequency of exposure tasks, cumulative duration, and timing relative to reproductive windows (preconception, implantation, first trimester, lactation)—informs the urgency and type of clinical response.

Fourth, where available from occupational health departments, quantitative workplace monitoring data (surface wipe sampling, air monitoring, biological monitoring) provide the most objective exposure characterization, although such data are rarely available in routine clinical settings [11,47,48]. Clinicians are encouraged to request occupational health consultation when quantitative data may influence clinical decisions.

Readers may refer to Table 2 to identify which studies employed more reliable exposure assessment methods (e.g., task-based modeling, biomonitoring) versus those relying solely on job-title classification, as this distinction directly affects the reliability of the reported risk estimates.

### 4.3. Preconception Counseling and Pregnancy-Sensitive Management

Preconception counseling should address specific occupational exposures (antineoplastic agents, HLDs, sterilants, formaldehyde, ethylene oxide, solvents) and work-organization factors before conception, as early embryonic development may occur before pregnancy recognition [17,26,27,28,29,42]. Workers actively trying to conceive, undergoing assisted reproductive treatment, pregnant, or breastfeeding may require individualized task modification when controls are inadequate or direct handling of hazardous drugs or highly reactive sterilants is unavoidable.

Lactation-period assessment should be handled cautiously. Evidence on lactational transfer of specific occupational toxicants remains limited, but return-to-work conditions, shift work, and chemical tasks may affect breastfeeding duration and maternal health [1,29]. Clinical follow-up should therefore consider both reproductive outcomes and work-organization factors.

#### Lactation Period and Postpartum Return-to-Work Considerations

The postpartum return-to-work period deserves particular clinical attention because many healthcare workers resume tasks involving hazardous drugs, high-level disinfectants, or shift work while breastfeeding. However, direct evidence on lactational transfer of occupational reproductive toxicants remains extremely limited.

For antineoplastic agents, data on breastmilk contamination from occupational (as opposed to therapeutic) exposure are restricted to isolated case reports and animal models; no prospective human studies have quantified occupational antineoplastic concentrations in breastmilk under workplace exposure conditions. Similarly, evidence on lactational transfer of formaldehyde, glutaraldehyde, ortho-phthalaldehyde (OPA), and ethylene oxide in occupational settings is sparse, with most available toxicological data derived from environmental or industrial exposure scenarios rather than healthcare-specific contexts.

The NIOSH reproductive health guidance identifies lactational transfer of occupational toxicants as an important evidence gap and recommends precautionary measures for breastfeeding workers handling hazardous drugs, particularly those classified as NIOSH Group 1 [29]. The paucity of human data in this domain represents a critical research gap that limits evidence-based clinical guidance.

In the absence of definitive evidence, clinicians should apply the precautionary principle for breastfeeding healthcare workers, while avoiding unnecessary work restrictions that may adversely affect breastfeeding duration and maternal wellbeing. Practical clinical considerations for the return-to-work period include: (1) task reassignment options to minimize direct handling of hazardous drugs or highly reactive sterilants during breastfeeding; (2) scheduling accommodations to support milk expression and minimize fatigue-related risks from shift work; (3) occupational health consultation to develop individualized return-to-work plans; and (4) reassessment of exposure controls before, rather than after, the worker resumes hazardous tasks. These recommendations are classified as Tier 3 (expert opinion) given the limited evidence base.

### 4.4. Clinical Surveillance and Preventive Framework

Effective prevention requires communication among the worker, occupational physician, obstetrician or gynecologist, supervisor, pharmacy leadership, and occupational safety team. Recommendations should be linked to actual exposure conditions, including availability and use of CSTDs, biological safety cabinets, local exhaust ventilation, automated reprocessing systems, appropriate gloves, respiratory protection, spill management, and surface or biological monitoring [3,10,11,24,25,31,36,45,47,48,49].

## 5. Limitations of the Current Evidence

Several important limitations should be acknowledged, and their implications for the interpretive framework of this review warrant explicit discussion. First, the evidence base is highly heterogeneous in study design, population, exposure assessment methodology, outcome definition, and adjustment strategy. Most included primary studies are observational, frequently retrospective, and heavily reliant on self-reported occupational exposure assessment rather than quantitative measurement. This predominantly observational evidence base limits causal inference across all exposure categories reviewed and prevents direct comparison across findings [1,2,4,5,7,14,15,16,17,18,19,20,21,22,23,33,34,35,41,42,43].

Second, many studies rely on job title, department assignment, or self-reported task history rather than repeated quantitative exposure assessment. Broad occupational titles do not accurately capture task-level exposure variability (see Section 4.2) [5,7,12,16,33,35,36,42,47,48]. Third, reproductive outcomes such as early miscarriage and time to pregnancy are susceptible to recall bias, selection bias, confounding by age and prior reproductive history, and the healthy worker effect [2,4,17,18,19,20,21,22].

Fourth, publication bias may overrepresent positive findings, as studies reporting null associations with occupational exposure may be less likely to be published.

Fifth, the present review is narrative rather than systematic. Accordingly, this review was not registered in PROSPERO, which accepts only systematic review registrations. Although we employed several methodological safeguards to enhance transparency—including a predefined PECO framework (Table 1), an adapted PRISMA-style flow diagram for transparency (Figure 1), SANRA self-assessment (Supplementary Table S1), and a modified evidence-level classification (Table 2)—we did not conduct formal risk-of-bias assessment using standardized tools (e.g., ROBINS-I, Newcastle–Ottawa Scale) or quantitative synthesis. As discussed in Section 2.5, the heterogeneity in exposure assessment modalities, outcome ascertainment, and study populations precluded quantitative pooling [2,14,17,18,30]. Where robust meta-analytic evidence already exists for specific agent–outcome pairs, we cite those pooled estimates directly.

Sixth, the evidence-level classification applied in Table 2 provides a hierarchy-based reader aid but does not replace formal quality appraisal; readers should interpret individual study findings with attention to sample size, exposure measurement validity, and potential confounding.

Seventh, the translation of this framework into routine clinical workflows may be constrained by time limitations during patient encounters, incomplete integration of occupational history modules into electronic medical records, and limited training on occupational reproductive hazards among obstetrics and gynecology providers. Future studies should evaluate the uptake and clinical impact of structured occupational history-taking tools such as those proposed in this review.

## 6. Future Directions

Evidence remains strongest for nurses and antineoplastic agents, while laboratory personnel, sterilization technicians, environmental services workers, pathology staff, and other healthcare workers remain less comprehensively studied [2,6,9,29,50].

Future research should prioritize prospective designs with standardized reproductive outcome definitions (e.g., clinically confirmed versus self-reported miscarriage), quantitative exposure measurements incorporating task-based assessment and biomonitoring where feasible, and sex-disaggregated analyses. Intervention studies evaluating the effectiveness of exposure controls (e.g., CSTDs, ventilation upgrades, automated reprocessing) on reproductive outcomes are particularly needed. Additionally, studies should examine whether structured occupational history-taking tools—such as the clinical questions proposed in Table 3—improve detection of workplace reproductive hazards in prenatal and preconception care settings. Research extending beyond nursing populations to include sterilization technicians, pathology staff, laboratory personnel, and environmental services workers would address important evidence gaps identified in this review.

## 7. Conclusions

Occupational exposure to reproductive toxicants, particularly antineoplastic agents, represents a clinically relevant, biologically plausible, and often under-recognized concern for women healthcare workers (Tier 1: evidence-supported). Evidence for HLDs and sterilants suggests precautionary risk signals warranting exposure-control verification but remains substantially more heterogeneous and context-dependent (Tier 2: precautionary) [5,7,14,16,17,18,23,34,35,42]. Work-organization factors contribute additional risk modifiers supported primarily by observational evidence (Tier 3: expert opinion). Formaldehyde and ethylene oxide require targeted attention in pathology, anatomy, sterilization, and reprocessing settings [6,8,26,27,28,29].

**Figure 2:**
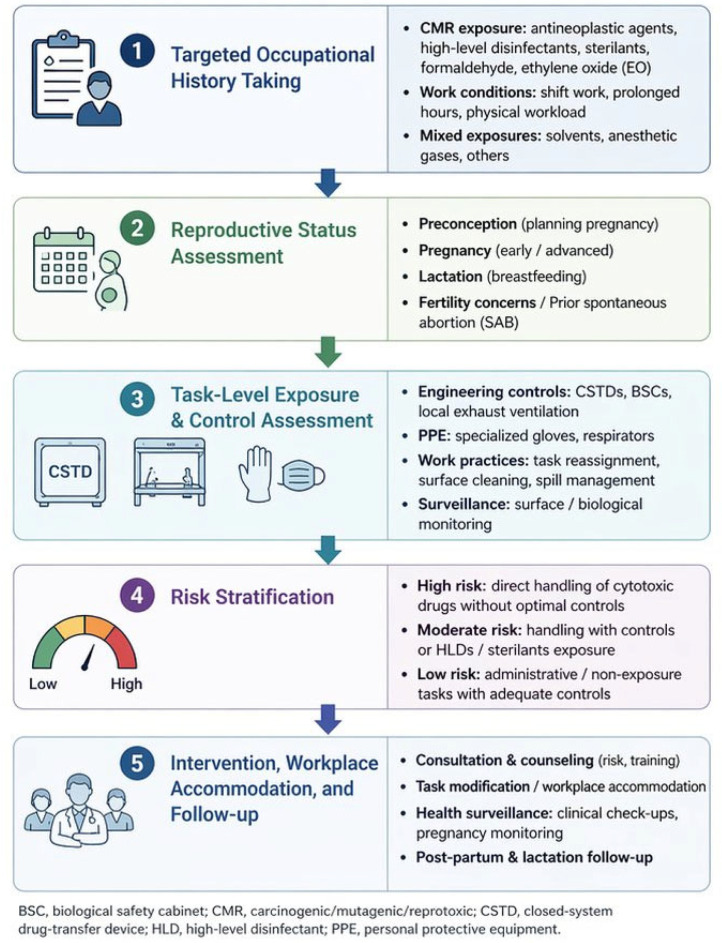
Proposed clinical decision pathway for reproductive health surveillance among women healthcare workers. This pathway is intended as a clinical decision support tool, not a validated clinical guideline. Recommendation strength varies by exposure category: Tier 1 (evidence-supported) for antineoplastic agents; Tier 2 (precautionary) for HLDs and sterilants; Tier 3 (expert opinion) for mixed exposures and work-organization factors. The pathway has not been externally validated and should be interpreted as a clinical education tool pending further research.

**Figure 3:**
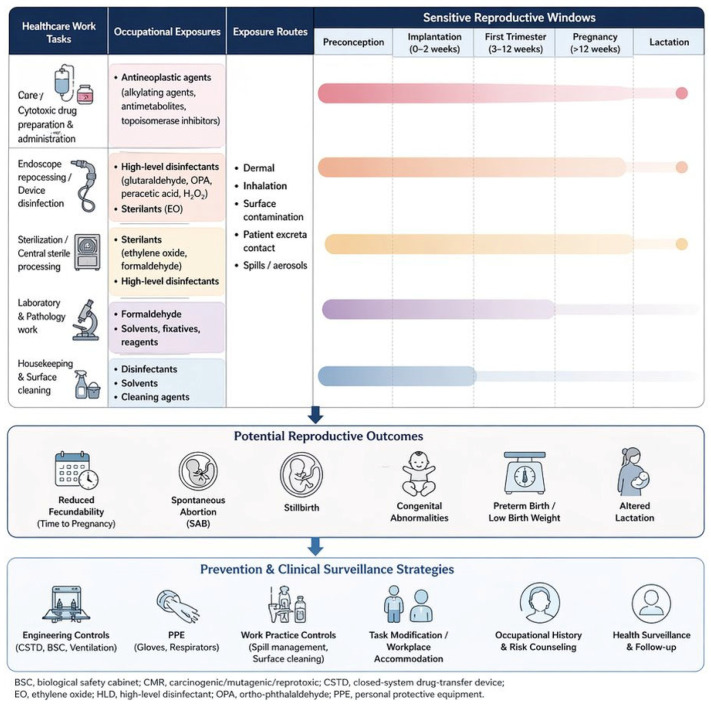
Proposed integrated framework linking occupational exposures, biological plausibility, reproductive outcomes, and preventive actions. The strength of evidence varies substantially across exposure categories: antineoplastic agents have the strongest epidemiological support (Tier 1), HLDs and sterilants occupy an intermediate position (Tier 2), and work-organization factors are supported primarily by observational evidence with inconsistent findings (Tier 3). This framework has not been externally validated and should be interpreted as a clinical education tool pending further research.

Preventing adverse reproductive outcomes requires coordinated clinical and occupational action, with obstetrician-gynecologists, primary care clinicians, and reproductive endocrinologists well-positioned to incorporate occupational assessment into routine care. We recommend that task-specific occupational history taking (Table 3; Figure 2) become a standard element of preconception and early pregnancy visits to triage the need for exposure-control verification, workplace accommodation, or occupational medicine referral. The integrated prevention framework (Figure 3, Table 4) may further support communication among clinical, occupational, and administrative teams. Clinicians should recognize that the evidence base informing these recommendations is predominantly observational and subject to self-report bias, exposure misclassification, healthy worker effects, and publication bias. Recommendations should therefore be calibrated to the strength of the underlying evidence, applying the precautionary principle where warranted while avoiding overstatement of causal certainty. By applying NIOSH, USP <800>, ASHP, and related safety frameworks to individual workplace tasks, clinicians can support timely exposure reduction and reproductive-health-sensitive prevention without overstating causality from heterogeneous evidence [3,10,24,25,31].

## Figures and Tables

**Table 1:** PECO framework for literature selection.

PECO Element	Inclusion Criteria	Exclusion Criteria
Population (P)	Women healthcare workers (nurses, pharmacists, laboratory personnel, sterile processing technicians, pathology staff, hospital housekeeping, operating room personnel)	General population without occupational healthcare exposure; male-only study populations; animal-only studies
Exposure (E)	Occupational exposure to antineoplastic agents, hazardous drugs, high-level disinfectants (glutaraldehyde, OPA, peracetic acid, H_2_O_2_), sterilants (ethylene oxide, formaldehyde), solvents, anesthetic gases, or work-organization factors (shift work, prolonged hours, physical workload)	Non-occupational environmental exposure; dietary or lifestyle-only exposures; therapeutic patient exposure
Comparison (C)	Unexposed healthcare workers, general female population, or within-cohort comparisons by exposure level/duration (where available)	No comparator required for descriptive studies or guidelines; absence of comparator did not lead to exclusion
Outcome (O)	Reproductive and pregnancy outcomes: spontaneous abortion, stillbirth, congenital abnormalities, reduced fecundability/time to pregnancy, preterm birth, low birth weight, breastfeeding disruption; occupational cancer risk where reproductively relevant	Non-reproductive occupational outcomes (e.g., musculoskeletal injury, respiratory symptoms without reproductive relevance); patient treatment outcomes

**Table 2:** Characteristics, evidence level, exposure assessment, and main findings of key studies.

Year	First Author	Design/Evidence Level	Exposure Assessment	Main Outcome	Principal Finding Effect Estimate	Key Limitation
1990	Stucker [34]	Exposed–unexposed/III	Job records; perfusion task logs	Spontaneous abortion	Spontaneous abortion (SAB): 15.7% exposed vs. 10.5% unexposed (*p* < 0.05)	Small sample; limited exposure quantification
1999	Valanis [23]	Retrospective survey/III	Self-reported drug handling	Miscarriage, stillbirth	SAB OR range 1.5–2.3 among handling mixing nurses	Recall bias; self-report
2007	Fransman [35]	Cohort + task-based model/II	Dermal exposure estimation from task questionnaire	TTP, preterm birth, LBW	Highest dermal exposure: FR 0.8 (95% CI: 0.6–1.0) for TTP; preterm OR 1.7	Modeled exposure; retrospective fertility data
2010	Ratner [33]	Historical cohort + registry/II	Department-level exposure classification	Cancer incidence, pregnancy outcomes	Breast cancer SIR 1.83 (95% CI: 1.03–3.23); pregnancy outcomes NS	Ecological exposure; healthy worker effect
2012	Lawson [16]	NHS II analysis/II	Self-reported occupational exposure	Spontaneous abortion	Antineoplastic → SAB aOR ≈ 2.0; nulliparous aOR 3.5; sterilizing agents → late SAB aOR ≈ 2.0	Self-report; residual confounding
2017	Gaskins [5]	Prospective cohort (NHS3)/II	Self-reported HLD use frequency	Fecundability (TTP)	HLD use: FR 0.87 (95% CI: 0.77–0.98); attenuated with PPE	Exposure intensity not fully quantified
2019	Nassan [41]	Prospective cohort (NHS3)/II	Self-reported antineoplastic administration	Fecundability	FR ≈ 1.0 (NS) when exposure controls consistently used	Self-report; limited dose estimation
2021	Nassan [42]	Prospective cohort (NHS3)/II	Self-reported prepregnancy handling	Miscarriage	AD handling: HR 1.26 (95% CI: 0.97–1.64); inconsistent controls: HR 1.46 (95% CI: 1.02–2.10)	Self-report; unmeasured confounders
2021	Ding [7]	Prospective cohort (NHS3)/II	Self-reported HLD use	Miscarriage	Overall HR ≈ 1.0 (NS); within 12 months: HR elevated (borderline significant)	Exposure window definition; self-report
2023	Liu [14]	SR + meta-analysis/I	Pooled from 11 heterogeneous studies	SAB, stillbirth, congenital abnormalities	Pooled OR: SAB, stillbirth, congenital abnormalities all statistically significant	Heterogeneous exposure assessment across studies
2023	Marsters [2]	Scoping review/I	Mapped 98 studies by hazard type	Mixed pregnancy outcomes	No pooled estimate; elevated SAB signals across studies	Heterogeneous designs; limited to physician-related hazards
2024	Izadi [1]	Cross-sectional/IV	Questionnaire-based	Multiple reproductive outcomes	Chemical exposure, shift work, psychiatric factors: *p* < 0.05 for selected outcomes	Cross-sectional design; temporal ambiguity; self-report

Evidence levels adapted from the Oxford Centre for Evidence-Based Medicine (OCEBM) hierarchy [40]: I = systematic review/meta-analysis; II = prospective cohort; III = retrospective cohort/case–control; IV = cross-sectional/case series; V = narrative review/expert opinion/guideline. LBW, low birth weight; NHS, Nurses’ Health Study; NS, not statistically significant; SAB, spontaneous abortion; SR, systematic review; TTP, time to pregnancy.

**Table 3:** Clinical risk questions for women healthcare workers.

Domain	Clinical Question	Clinical Interpretation
Antineoplastic agents	Do you actively prepare, administer, dispose of, or clean spills involving chemotherapy or hazardous drugs?	Identifies direct hazardous-drug exposure and need for task review.
Surface contamination	Do you handle contaminated tubing, linens, patient excreta, or drug-contaminated surfaces?	Captures indirect dermal/oral transfer risks that may be missed by job title.
HLDs and sterilants	Do you use glutaraldehyde, OPA, formaldehyde, ethylene oxide, peracetic acid, or automated reprocessors?	Identifies device-reprocessing, sterilization, and pathology-related exposure.
Exposure controls	Are CSTDs, biological safety cabinets, local exhaust ventilation, double gloves, gowns, and spill protocols used consistently?	Evaluates risk mitigation and whether workplace accommodation is needed.
Reproductive status	Are you planning pregnancy, pregnant, undergoing fertility treatment, postpartum, or breastfeeding?	Determines the urgency and type of counseling or task modification.
Work organization	Do you work night shifts, extended hours, heavy lifting, or high-stress duties?	Integrates chemical and non-chemical reproductive risk factors.

**Table 4:** Clinical and occupational prevention framework.

Exposure Source	Primary Clinical Concern	Workplace Control Mechanism	Required Clinical Action	Evidence Tier
Antineoplastic agents	Miscarriage, stillbirth, congenital abnormalities, genotoxicity, cancer concerns	CSTDs, biological safety cabinets, PPE, spill protocols, surface monitoring	Preconception counseling, task review, accommodation if controls are inadequate	Tier 1 (Evidence-supported)
HLDs	Reduced fecundability, miscarriage risk signals	Local exhaust ventilation, automated reprocessors, substitution, gloves, eye/respiratory protection	Time-to-pregnancy history, PPE adherence assessment, ventilation review	Tier 2 (Precautionary)
Formaldehyde/ethylene oxide	Carcinogenic and reproductive concerns	Enclosure, air monitoring, substitution, fume hood/ventilation	Pregnancy-sensitive restriction where exposure is uncontrolled; symptom/exposure review	Tier 2 (Precautionary)
Mixed hazards	Preterm birth, pregnancy loss, lactation disruption, fatigue-related risks	Scheduling modification, ergonomic controls, staffing support	Whole-work assessment, fetal growth and maternal health surveillance	Tier 3 (Expert opinion)

## Data Availability

Data sharing is not applicable to this article as no new data were created or analyzed in this study.

## Supplementary Materials

The following supporting information can be downloaded at: https://www.mdpi.com/article/10.3390/jcm15124651/s1, Table S1: SANRA self-assessment for this narrative review; Table S2: Database-specific search strategies.

## Abbreviations

BSC	Biological safety cabinet
CMR	Carcinogenic/mutagenic/reprotoxic
CSTD	Closed-system drug-transfer device
EO	Ethylene oxide
FR	Fecundability ratio
HLD	High-level disinfectant
H_2_O_2_	Hydrogen peroxide
LBW	Low birth weight
NHS	Nurses′ Health Study
NS	Not statistically significant
OCEBM	Oxford Centre for Evidence-Based Medicine
OPA	Ortho-phthalaldehyde
PECO	Population–Exposure–Comparison–Outcome
PPE	Personal protective equipment
SAB	Spontaneous abortion
SALSA	Search–Appraisal–Synthesis–Analysis
SANRA	Scale for the Assessment of Narrative Review Articles
SIR	Standardized incidence ratio
SR	Systematic review
TTP	Time to pregnancy
USP	United States Pharmacopeial Convention
