# Supplementary material for: Occupational Reproductive Health Risks Among Women Healthcare Workers: A Narrative Review for Clinical Surveillance, Preconception Counseling, and Prevention
Source: J Clin Med. 2026 Jun 15;15(12):4651. doi: 10.3390/jcm15124651 (PMC13301733; doi:10.3390/jcm15124651)
Supplement: Supplementary file 1 [file jcm-15-04651-s001.zip › Supplementary_Table_S1_SANRA.pdf]

Supplementary Table S1. SANRA Self-Assessment for This Narrative Review

SANRA Item	Score (0–2)	Justification
1. Justification of the article's importance	2	The introduction establishes clinical relevance of occupational reproductive hazards for women healthcare workers, identifies a gap in existing reviews (single-agent focus without clinical decision-making frameworks), and provides explicit justification for a narrative synthesis rather than meta-analysis (Section 1, paragraphs 4–5).
2. Statement of concrete aims or formulation of questions	2	The aims are explicitly stated in the final paragraph of the Introduction: to synthesize evidence on antineoplastic agents, HLDs, sterilants, and work-organization factors, and to translate this evidence into a clinically applicable framework for occupational history taking, preconception counseling, risk stratification, and surveillance. A PECO framework (Table 1) further operationalizes the review question.
3. Description of the literature search	2	Section 2 describes databases searched (PubMed, Scopus, Web of Science, Embase), search terms, PECO eligibility criteria, search period (through January 2025, updated March 2026), and the literature selection process with a PRISMA-informed flow diagram (Figure 1). Database-specific search strategies are provided in Supplementary Table S2. Approximate screening numbers are reported (320 identified → 285 screened → 85 assessed → 49 included).
4. Referencing	2	Fifty references are cited, including landmark cohort studies (Stucker 1990, Valanis 1999, Fransman 2007), recent prospective evidence (Nurses' Health Study 3 analyses: Gaskins 2017, Nassan 2019/2021, Ding 2021), meta-analyses (Liu 2023, Quansah 2010), scoping reviews (Marsters 2023), and major guideline documents (NIOSH 2024, USP <800>, ASHP 2018, Cochrane 2018). Evidence levels (OCEBM I–V) are classified for key studies in Table 2.
5. Scientific reasoning	2	Evidence is organized by exposure category (antineoplastic agents, HLDs/sterilants, mixed/work-organization) with attention to biological plausibility (Section 3.2.1), epidemiological consistency and inconsistency (Sections 3.2.2, 3.3), exposure–control modification of risk (Nassan 2019 vs. 2021), and appropriate hedging throughout. The review distinguishes between levels of evidence strength across exposure categories and explicitly acknowledges where evidence is heterogeneous (HLDs) versus more consistent (antineoplastic agents).
6. Appropriate presentation of data	2	Four tables summarize PECO criteria (Table 1), key study characteristics with evidence levels and effect estimates (Table 2), clinical risk questions (Table 3), and the prevention framework (Table 4). Three figures present a PRISMA-informed flow diagram (Figure 1), a clinical decision pathway (Figure 2), and an integrated exposure–outcome–prevention framework (Figure 3). A "What This Review Adds" box highlights the novel contributions.

SANRA = Scale for the Assessment of Narrative Review Articles [16]. Each item scored 0 (low standard), 1 (moderate standard), or 2 (high standard). Total score: 12/12.

Reference: Baethge, C.; Goldbeck-Wood, S.; Mertens, S. SANRA—a scale for the quality assessment of narrative review articles. Res. Integr. Peer Rev. 2019, 4, 5. doi:10.1186/s41073-019-0064-8.
