# Supplementary material for: Occupational Reproductive Health Risks Among Women Healthcare Workers: A Narrative Review for Clinical Surveillance, Preconception Counseling, and Prevention
Source: J Clin Med. 2026 Jun 15;15(12):4651. doi: 10.3390/jcm15124651 (PMC13301733; doi:10.3390/jcm15124651)
Supplement: Supplementary file 1 [file jcm-15-04651-s001.zip › Supplementary_Table_S2_Search_Strategy.pdf]

Supplementary Table S2. Database-specific search strategies

Database	Search Date	Search String (abbreviated)	Filters Applied	Records Identified
PubMed/MEDLINE	2025-01-15; updated 2026-03-10	(healthcare workers OR nurses OR pharmacists OR hospital staff) AND (antineoplastic agents OR hazardous drugs OR chemotherapy) AND (pregnancy outcomes OR miscarriage OR spontaneous abortion OR fecundity OR reproductive outcomes)	English; Human; Journal Article, Review, Systematic Review, Meta-Analysis	142
Scopus	2025-01-15; updated 2026-03-10	TITLE-ABS-KEY((healthcare worker* OR nurs*) AND (disinfectant* OR sterilant* OR formaldehyde OR ethylene oxide) AND (reproduct* OR pregnan* OR fecund* OR miscarriage))	Article, Review; English	78
Web of Science	2025-01-15; updated 2026-03-10	TS=((healthcare worker* OR nurs* OR pharmacist*) AND (antineoplastic OR hazardous drug* OR high-level disinfectant*) AND (reproductive outcome* OR pregnancy OR spontaneous abortion OR infertility))	Article, Review; English	65
Embase	2025-01-15; updated 2026-03-10	(healthcare worker OR nurse OR pharmacist) AND (antineoplastic agent OR hazardous drug OR disinfectant OR sterilant) AND (pregnancy outcome OR miscarriage OR fecundity OR congenital abnormality) AND ('article'/it OR 'review'/it)	English; Human	35
Additional sources	2025-01-15; updated 2026-03-10	NIOSH, USP, CDC/NIOSH, ASHP, Cochrane guideline documents; reference list scanning of included studies	—	45

Total records identified from databases: 320. Additional records from guidelines and reference lists: 45. After deduplication: 285 unique records screened.
